# Reduced miR-338-3p contributes to polycystic ovarian syndrome by inhibiting proliferation and enhancing apoptosis

**DOI:** 10.1186/s41065-025-00498-1

**Published:** 2025-07-12

**Authors:** Lin Liang, Jie Lv, Wei Li, Chengwen Song, Ying Chen, Huafang Wei

**Affiliations:** Department of Gynecology, General Hospital of Central Theater Command of Chinese People’s Liberation Army, 627 Wuluo Road, Wuchang District, Hubei Wuhan, 430064 China

**Keywords:** Polycystic ovarian syndrome, miR-338-3p, Apoptosis, Proliferation

## Abstract

**Background:**

Polycystic ovarian syndrome (PCOS) is a frequently occurring disorder affecting reproductive and metabolic health. miR-338-3p is implicated in early follicular development. We aimed to investigate the expression of miR-338-3p in PCOS patients and its effects on proliferation and apoptosis of ovarian granulosa cells.

**Methods:**

The study included 100 healthy women and 110 women diagnosed with PCOS as participants. Reverse transcription quantitative PCR (RT-qPCR) was used to detect the expression of miR-338-3p and phosphatase and tensin homolog (PTEN), and receiver operating characteristic (ROC) was employed to evaluate the diagnostic efficacy of miR-338-3p. Cell proliferation and apoptosis were detected by Cell Counting Kit-8 (CCK-8) and flow cytometry. Pearson correlation analysis was used to assess the correlations between miR-338-3p and luteinizing hormone (LH), testosterone, or PTEN. The target relationship of miR-338-3p and PTEN was confirmed via dual-luciferase assay.

**Results:**

Serum miR-338-3p was decreased in PCOS patients, and it was negatively correlated with both LH and testosterone. Downregulation of miR-338-3p inhibits the proliferation of ovarian granulosa cells and enhances cell apoptosis, whereas upregulation produces the opposite effect. PTEN inhibition subverted the inhibited proliferation and enhanced apoptosis regulated by miR-338-3p inhibitor.

**Conclusions:**

Reduced miR-338-3p levels have potential predictive value in distinguishing individuals with PCOS patients from normal population. Mechanistically, this microRNA regulates the PTEN gene to inhibit granulosa cell proliferation and promote apoptosis, thereby contributing to the pathological processes of PCOS.

## Background

Polycystic ovary syndrome (PCOS) is a widespread condition characterized by disruptions in reproductive endocrine function and metabolic balance among women of reproductive age [1]. Women with PCOS during their reproductive years often face risks such as infertility, gestational diabetes, pregnancy-induced hypertension, as well as preterm birth [2, 3]. In the long term, patients with PCOS are also at risk for multiple health issues, including cardiovascular disease, endometrial cancer, and type 2 diabetes [4]. It has been reported that PCOS affects 5–20% of women in their reproductive years globally [5]. Despite links to genetics, environment, and lifestyle, the exact etiology of PCOS remains unclear and there is currently no cure for the condition [6]. Thus, understanding the pathogenesis of PCOS is crucial for developing effective treatments.

MicroRNAs (miRNAs) are small molecules that control gene activity by binding to target mRNAs, thereby affecting cell function and disease progression [7, 8]. Current reports have underscored the crucial role of miRNAs in the pathogenesis of PCOS. For instance, miR-222, miR-146a, as well as miR-30c are proved to be enhanced in patients with PCOS. Moreover, serum miR-222 correlates positively with serum insulin levels, while miR-146a is linked to lower testosterone levels. These miRNAs can serve as biomarkers for PCOS [9]. In the granulosa cells of individuals with PCOS, the increased miR-93-5p has been demonstrated to drive the apoptosis as well as ferroptosis by NF-κB pathway [10]. Some studies manifest that the exosomal miR-143-3p and miR-155-5p are involved in the regulation of follicular dysplasia in PCOS by modulating glycolysis [11]. Additionally, in PCOS, the low expression of miR-96-5p is manifested to inhibit granulosa cell proliferation and the synthesis of estrogen by targeting FOXO1 [12].

Insulin resistance (IR) is a significant component of the core pathophysiological mechanisms underlying PCOS [13]. The hyperinsulinemia caused by IR can trigger a cascade of metabolic disturbances such as elevated blood glucose levels and dyslipidemia, worsening the PCOS symptoms and elevating the likelihood of developing metabolic disorders, such as type 2 diabetes and cardiovascular disease [14]. It also stimulates ovarian androgen production, disrupting follicle development and ovulation [15]. Existing reports have shown the significant role of miR-338-3p in IR. As reported, it is decreased in IR mouse and cell models, disrupting hepatic insulin signaling via PP4 and impairing glycogen synthesis [16]. Results from hepatocytes indicate that miR-338-3p also contributes to gluconeogenesis [17]. Moreover, it affects early follicle development and ovarian function by interfering with granulosa cell proliferation and estradiol production [18]. Thus, miR-338-3p might be linked to the pathogenesis of PCOS.

This study examined the expression profile of miR-338-3p in patients with PCOS and elucidated its impact on the proliferation and apoptosis of ovarian granulosa cells.

## Methods

### Study object and serum preparation

All participants in this study were from General Hospital of Central Theater Command of Chinese People’s Liberation Army, comprising 100 healthy women and 110 women diagnosed with PCOS. The identification of PCOS was determined by the Rotterdam Criteria established in 2003. Exclusion criteria included: (1) use of hormonal medications within the past 3 months; (2) other conditions causing similar symptoms, such as Cushing’s syndrome, congenital adrenal hyperplasia, or androgen-secreting tumors; (3) presence of other ovarian diseases or severe systemic diseases; (4) age more than 18 years. The control group consisted of healthy women with normal menstrual cycles, ovary function, and no gynecological diseases, and were matched with the PCOS group in terms of age and BMI. Furthermore, the healthy individuals also took no hormonal medication use in the past 3 months. All subjects were Han Chinese females. Venous blood was collected in the morning following a 10-hour fast. The serum samples, obtained by centrifugation at 4000 rpm for 10 min, were stored at -80 °C.

All participants signed written informed consent forms before the start of the study, agreeing to the use of their clinical data and biological samples for scientific research. Approval was obtained from the ethics committee of General Hospital of Central Theater Command of Chinese People’s Liberation Army. The procedures used in this study adhere to the tenets of the Declaration of Helsinki.

### Hormone level analysis

Serum levels of follicle-stimulating hormone (FSH), luteinizing hormone (LH), oestradiol, and Testosterone were measured using an electrochemiluminescence immunoassay (ECLIA) with the Cobas e601 analyzer (Roche Diagnostics, Germany) according to the manufacturer’s instructions. Briefly, 100 µL of serum was added to the reaction cuvette, followed by the addition of specific labeled antibodies and magnetic beads. After incubation and washing, the chemiluminescent signal was detected and converted to hormone concentrations using pre-calibrated standard curves.

### Cell culture and transfection

The human ovarian granulosa cell line (KGN) was purchased from Wuhan PrimeGene Biotechnology Co., Ltd. (Product No.: CL-0603). The commercialized granulose cells were maintained in DMEM/F12 culture medium adding FBS (10%) with penicillin and streptomycin. granulosa cells were cultured in a sterile and humidified incubator with 5% CO₂. The complete medium was replenished every 48 h to sustain nutrient supply. For transient transfection, the FBS and antibiotics were removed. The miR-338-3p mimic (miR-mimic), miR-338-3p inhibitor (miR-inhibitor) and the corresponding negative control (miR-NC) were transported into cells via lipofectamine 3000 to mediate the expression level of miR-338-3p. Besides, PTEN knockdown was achieved by the small interfering RNA for PTEN (si-PTEN) with the negative control (si-NC).

### RT-qPCR

Sample Collection: For serum samples, 200 µL was transferred to RNase-free centrifuge tubes. Granulocytes were washed twice with pre-cooled PBS in culture dishes, then lysed with 1 mL TRIzol via pipetting. Hemolysis was assessed by color observation (pink/red hues) and spectrophotometric measurement at 414 nm (A414 > 0.2 as exclusion threshold). To validate 20-nucleotide RNA recovery, a synthetic oligonucleotide (10 fmol, 5’-GUUUGUGCUUAGGUUGUGGUU-3’) was spiked into samples before RNA extraction using mirVana Kit (Thermo Fisher Scientific, USA). RT-qPCR showed recovery rates of 85.6% ± 3.2% (serum, *n* = 5) and 88.3% ± 2.8% (granulocytes, *n* = 5), confirming efficient recovery.

RNA extraction from serum or cells was performed using the Trizol method (Thermo Fisher Scientific, USA). The RNA was then applied for subsequent reverse transcription. The mixture of reverse transcription was assembled on ice following the specifications, with the RNA samples being introduced last. Following the reverse transcription, the products underwent a thermal inactivation at 85 °C for 5 min and were subsequently stored at -20 °C. A quantitative PCR kit (Thermo Fisher Scientific, USA) incorporating SYBR Green Mix was employed to exam the miR-338-3p and PTEN levels. The reaction conditions are as follows: 95 ℃ for 10 min, 98 ℃ for 10 s, 59 ℃ for 30 s, 72 ℃ for 30 s, with a total of 40 cycles. For quantitative PCR, the expression level was assessed by 2^−△△CT^ method, while GAPDH was employed as a reference. Three technical replicates were set for each sample. The relevant primer sequences are as follows: miR-338-3p (Forward primer 5’- ATATCCTGGTGCTGAGTG − 3’, reverse primer 5’- GAACATGTCTGCGTATCTC − 3’); PTEN (Forward primer 5’-TCCCAGACATGACAGCCATC-3’, reverse primer 5’- TGCTTTGAATCCAAAAACCTTACT-3’); Reference gene GAPDH (Forward primer 5’- GGCACCCAGCACAATGAAG − 3’, reverse primer 5’- CCGATCCACACGGAGTACTTG-3’).

### Cell proliferation

The proliferative capacity of granulosa cells was assessed by CCK-8 kit. Granulosa cells in logarithmic growth phase were harvested to prepare a cell suspension, which was then seeded into a 96-well plate at 5 × 10^3^ cells for each well with 100µL culture medium. Following a designated period of treatment, 10µL of CCK-8 reagent was dispensed into each well and gently mixed to ensure uniform distribution. After incubation for 2 h, the absorbance was measured at 450 nm using a microplate reader. The proliferation rate was calculated relative to the miR-NC control group, with blank wells (media only) used to correct for background absorbance. Experiments were performed in 3 independent biological replicates, each with 3 technical replicates per group (total *n* = 9 per condition).

### Cell apoptosis

Cell apoptosis was analyzed using an Annexin V-FITC/PI Apoptosis Detection Kit (BD Biosciences, USA). Briefly, cells were harvested by centrifugation at 1500 rpm for 5 min, washed twice with cold PBS, and resuspended in 1× binding buffer at a concentration of 1 × 10⁶ cells/mL. Subsequently, 5 µL of Annexin V-FITC and 5 µL of PI were added, and cells were incubated in the dark at room temperature for 15 min. Samples were analyzed within 1 h using a flow cytometer (BD FACSCanto II), and data were processed with FlowJo software.

### Dual-luciferase reporter assay

The sequence containing the miR-338-3p binding site (AGCUUGC) in the 3’ untranslated region (UTR) of PTEN was amplified and cloned into the pmirGLO vector. Wild-type luciferase (WT-PTEN) and mutant (MUT-PTEN) vectors were generated by mutating the seed site to UCGAAAC using the Fast Site-Directed Mutagenesis Kit (TIANGEN, Beijing) according to the manufacturer’s protocol. All constructs were verified by Sanger sequencing. The luciferase assay was performed in triplicate biological repeats (*n* = 3), with three technical replicates per group. Outliers were identified using Grubbs’ test (*P* < 0.05) and excluded from the final analysis. These constructs were then co-transfected with miR-inhibitor, miR-mimic, or miR-NC, respectively. Then 48 h following co-transfection, the luciferase activity was assayed.

### Statistical analysis

Data analysis in this study was performed by SPSS 21.0 as well as GraphPad Prism 7.0. For normally distributed continuous variables, independent samples t-test was used to compare two groups. For comparisons involving three or more groups, one-way analysis of variance (ANOVA) with Tukey’s post-hoc test was performed. The diagnostic efficacy of miR-338-3p for PCOS was assessed via constructing receiver operating characteristic (ROC) curves. To assess model generalizability, 10-fold cross-validation was performed, and the mean AUC across folds was calculated. Differences between the original and cross-validated AUC were tested using a paired t-test. Additionally, Pearson correlation analysis was utilized to investigate the associations of miR-338-3p with serum levels of LH, testosterone, as well as the target gene PTEN. A P value under 0.05 denoted a statistically significant distinction.

## Results

### Basic characteristics of participants

The basic characteristics of enrolled participants were presented in Table 1. In the PCOS group, the mean age and body mass index (BMI) of patients were 27.47 ± 3.00 years and 23.12 ± 3.51 Kg/m^2^ respectively. There were no statistically significant differences in age and BMI between the PCOS group and the control group (*P* > 0.05), indicating comparability. Hormonal assays indicated that, although the levels of FSH and oestradiol were marginally higher in the PCOS group than that of the control group, these differences were not statistically significant (*P* > 0.05). In stark contrast, the PCOS group exhibited a significant elevation in the average levels of LH and Testosterone compared with the control group (*P* < 0.001).

**Table 1:** Basic characteristics of participants

Characteristics	Control (*n* = 100)	PCOS (*n* = 110)	*P* values
Age, years	28.17 ± 3.12	27.47 ± 3.00	0.102
BMI, Kg/m2	22.57 ± 3.86	23.12 ± 3.51	0.282
FSH (IU/L)	6.20 ± 1.54	6.53 ± 1.42	0.102
LH (IU/L)	6.11 ± 2.75	11.26 ± 4.91	< 0.001
Oestradiol (pg/ml)	40.98 ± 12.86	43.88 ± 11.49	0.086
Testosterone (ng/dL)	26.39 ± 6.81	45.07 ± 11.01	< 0.001

PCOS, polycystic ovary syndrome, BMI, body mass index, FSH, follicle-stimulating hormone, LH, luteinizing hormone

### Relative expression and diagnosability of miR-338-3p

**Fig. 1:**
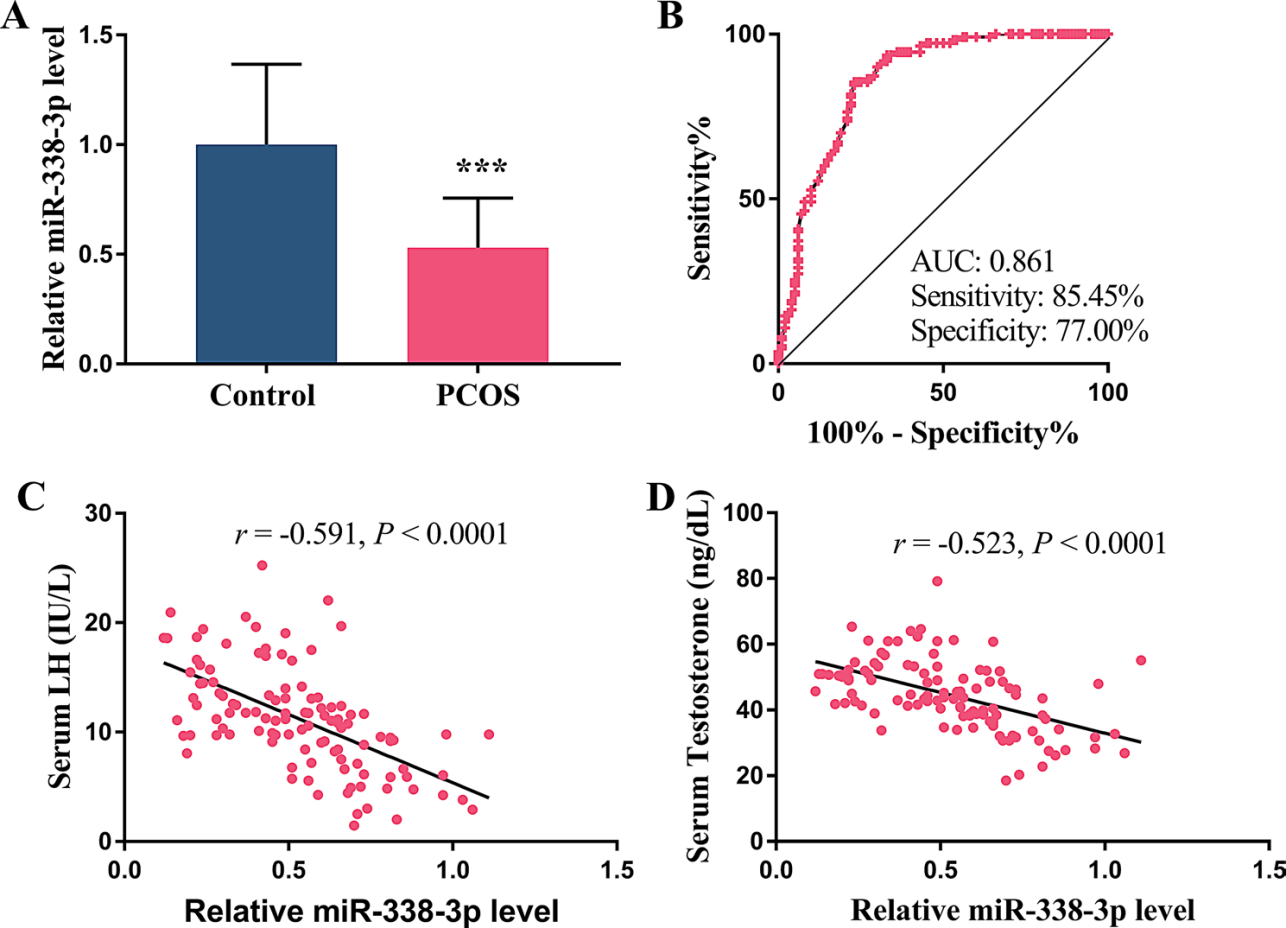
The predictive value of miR-338-3p and its correlation with clinical indicators of hormones. (**A**) Relative miR-338-3p level in patients with PCOS compared to the Control. (**B**) ROC curve of miR-338-3p. (**C**) Correlation of miR-338-3p level with serum LH. (**D**) Correlation of miR-338-3p level with serum testosterone. *** *P* < 0.001

The quantized analysis of miR-338-3p levels was conducted in patients with PCOS. Data confirmed a distinct reduction in the PCOS group by contrast with the control group (Fig. 1A, *P* < 0.001). The presence of significant statistical differences indicated a potentially strong association of miR-338-3p and PCOS. To further investigate its diagnostic potential, we constructed the ROC curve to assess the predictive performance of miR-338-3p for this disease. Analysis manifested that the AUC was 0.861 with a sensitivity of 85.45% and a specificity of 77.00%. The results of 10-fold cross-validation showed that the average AUC across all folds was 0.854 (95% CI: 0.821–0.887), which had no significant difference from the original AUC (*P* = 0.68, paired t-test) (Fig. 1B).

### Correlation of miR-338-3p with LH and testosterone

The potential correlation between miR-338-3p expression levels and the altered hormonal profiles in PCOS patients was examined using the Pearson correlation analysis. The results demonstrated a significant negative correlation between miR-338-3p and elevated serum LH, with a correlation coefficient (*r*) of -0.591 (Fig. 1C, *P* < 0.0001). Likewise, miR-338-3p was also confirmed to be negatively correlated with testosterone levels (*r* = -0.523, Fig. 1D, *P* < 0.0001).

### Effect of miR-338-3p on cell proliferation and apoptosis

**Fig. 2:**
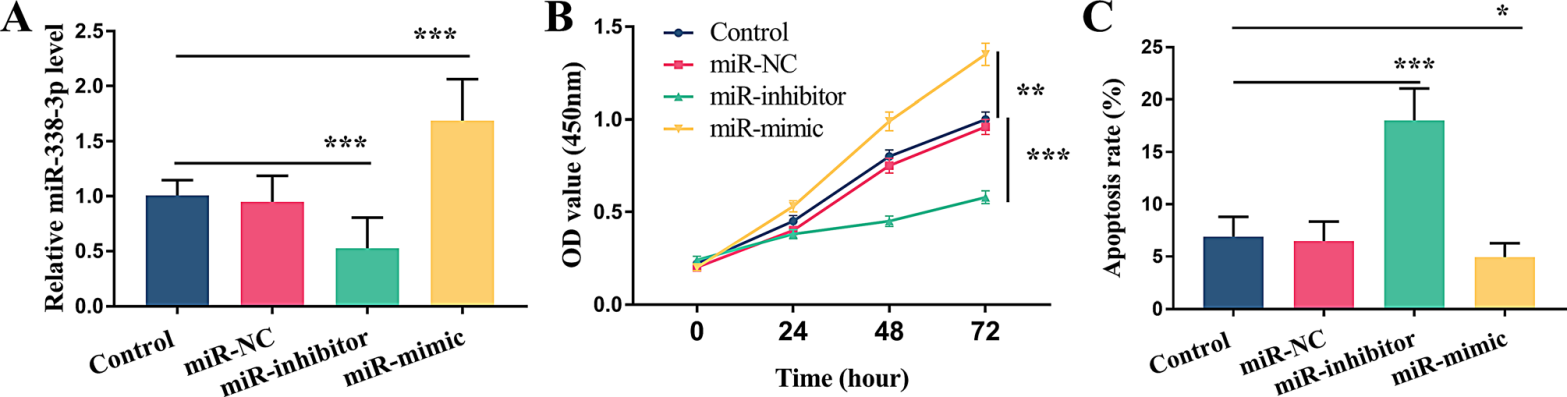
Effect of miR-338-3p on cell proliferation and apoptosis. (**A**) Relative expression of miR-338-3p. (**B**) The regulation of miR-338-3p level on cell viability. (**C**) The regulation of miR-338-3p level on apoptosis. * *P* < 0.05, ** *P* < 0.01, *** *P* < 0.001

Our previous quantitative PCR confirmed the low expression of miR-338-3p in PCOS. We then modulated miR-338-3p levels in granulosa cells through transfection to explore its effects on basic cell behavior. The artificial regulation of miR-338-3p expression in cells was achieved through transfection and verified by qPCR. To ensure the stability of miR-338-3p manipulation, we monitored its expression at multiple time points post-transfection. In KGN cells, miR-338-3p levels remained significantly elevated or suppressed at 24–72 h after transfection (Fig. 2A, *P* < 0.001). Functional assays showed that the cell proliferation rate in the miR-338-3p knockdown group was significantly lower than that in the control group (*P* < 0.001), while the cell proliferation rate in the miR-338-3p overexpression group was significantly higher than that in the control group (Fig. 2B, *P* < 0.01). Conversely, the opposite effects were observed in cell apoptosis. Downregulation of miR-338-3p significantly increased the apoptosis rate (*P* < 0.001), while upregulated of miR-338-3p effectively reduced the apoptosis of granulosa cells (Fig. 2C, *P* < 0.05).

### The potential target relationship of miR-338-3p with PTEN

**Fig. 3:**
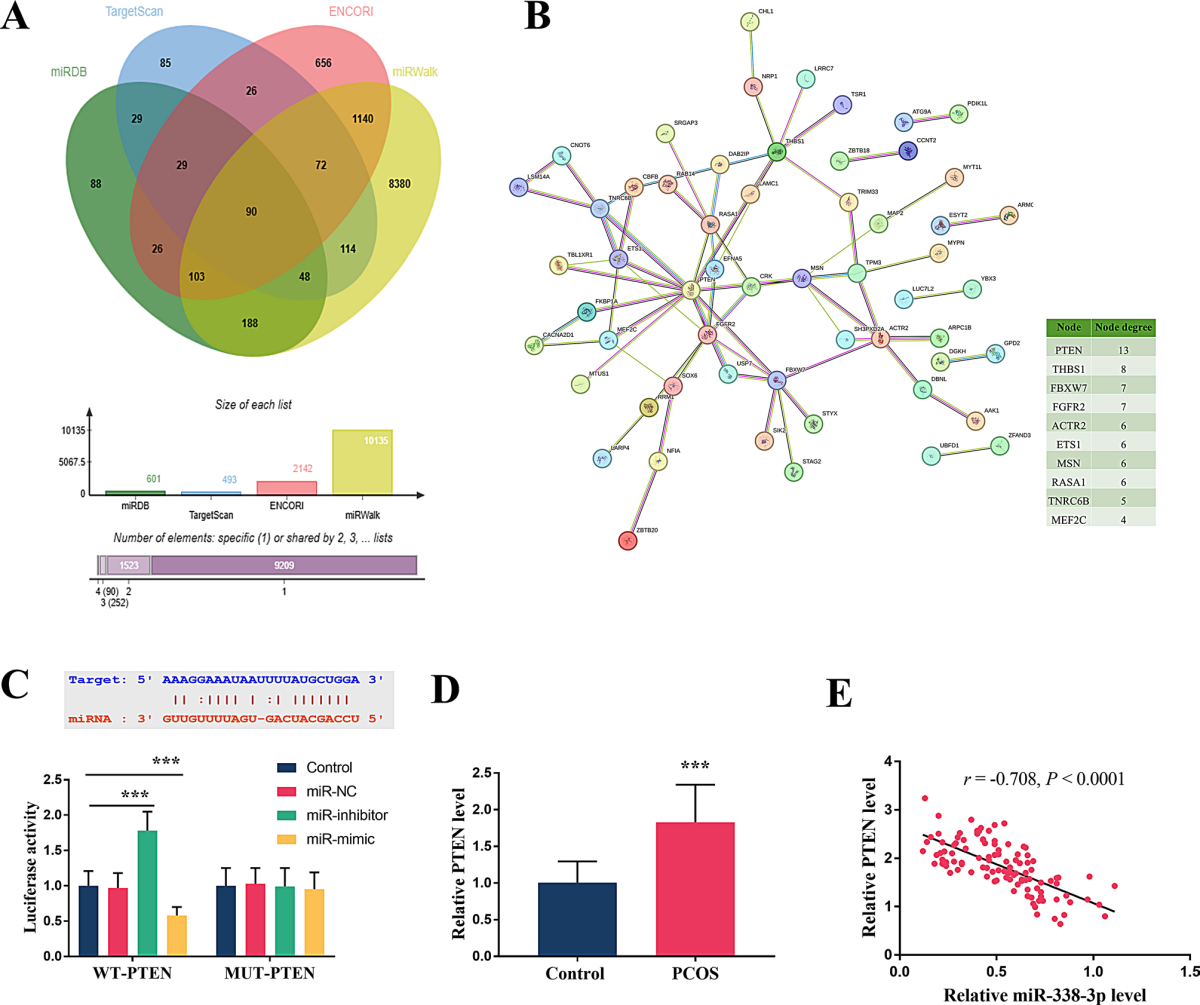
The underlying target relationship of miR-338-3p and PTEN. (**A**) Common elements predicted by different databases. (**B**) PPI network established by common elements. (**C**) Validation of the target relationship in dual-luciferase reporter assay. (**D**) Expression level of PTEN in patients with PCOS. (**E**) Correlation analysis of miR-338-3p and PTEN in patients with PCOS. *** *P* < 0.001

Databases including miRDB, TargetScan, ENCORI and miRWalk were used to predict the possible targets of miR-338-3p and 90 common genes were identified (Fig. 3A). The above shared genes were then subjected to the PPI network and a total of 10 predicted targets with high node degree were listed (Fig. 3B). Subsequent verification was conducted via the dual-luciferase assay, identifying PTEN as a potential target of miR-338-3p (Fig. 3C). In WT-PTEN group, the luciferase activity was visibly elevated by the miR-338-3p inhibition and suppressed by the miR-338-3p upregulation (Fig. 3C, *P* < 0.001). Moreover, no effect was observed in the MUT-miR-338-3p group. In the PCOS group, PTEN levels were found to be significantly elevated (Fig. 3D, *P* < 0.001). Further investigation revealed a negative correlation between PTEN expression and miR-338-3p levels (*r* = -0.708, *P* < 0.0001, Fig. 3E).

### Reversing effect of PTEN on cell proliferation regulated by miR-338-3p

**Fig. 4:**
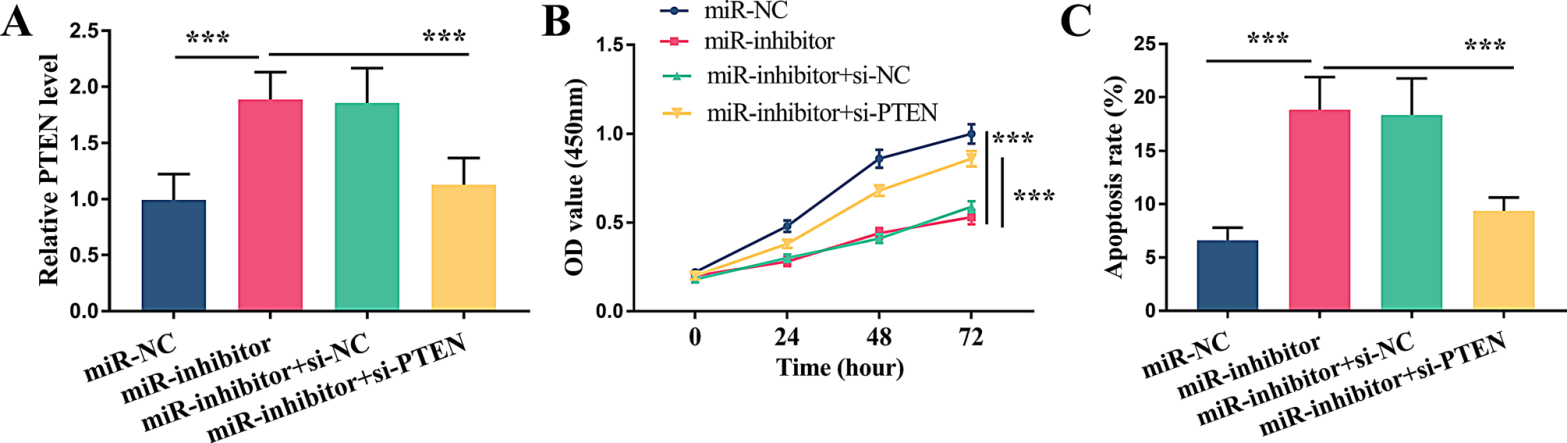
The negative regulation on PTEN by miR-338-3p. (**A**) Relative PTEN level. (**B**) The reversing effect of PTEN on cell proliferation regulated by miR-338-3p knockdown. (**C**) The reversing effect of PTEN on cell apoptosis regulated by miR-338-3p knockdown. *** *P* < 0.001

To further investigate the negative regulation between miR-338-3p and PTEN, we suppressed miR-338-3p expression in granulosa cells with inhibitor and observed a significant increase in PTEN levels (*P* < 0.001). Conversely, further knockdown of PTEN by siRNA transfection distinctly reduced its expression (Fig. 4A, *P* < 0.001). Cell proliferation assays showed that PTEN knockdown subverted the inhibitory effect of miR-338-3p downregulation (Fig. 4B, *P* < 0.001). Similarly, apoptosis analysis revealed that PTEN knockdown negated the pro-apoptotic effect of miR-338-3p downregulation, effectively (Fig. 4C, *P* < 0.001).

## Discussion

PCOS, a complex endocrine and metabolic disorder, is closely associated with gene regulatory networks mediated by miRNAs in its pathogenesis. This study systematically elucidates for the first time a novel mechanism by which miR-338-3p regulates the proliferation and apoptosis of ovarian granulosa cells by targeting the PTEN gene, providing critical physiological insights into the pathological process of PCOS.

Reviewing previous studies, the abnormally expressed miR-338-3p has been confirmed in a variety of diseases. For instance, in rodent models with IR as well as compensatory β-cell expansion, the expression of miR-338-3p is reported to be decreased [19]. During pregnancy and obesity, miR-338-3p is also observed to be reduced [20]. Additionally, research has shown that miR-338-3p is declined in both ovarian cancer tissues and cell lines [21]. Additionally, miR-338-3p may affect hormone levels by targeting specific genes in granulosa cells, inhibiting follicular development and hormone secretion, and thus raising LH and testosterone levels [18]. Similarly, this study found that serum miR-338-3p levels were significantly decreased in PCOS patients, and its expression was significantly negatively correlated with both LH and testosterone. However, these results only support a correlation, and the causal relationship between miR-338-3p and PCOS needs to be further explored through in vivo experiments. Studies have shown that miR-222 has good diagnostic efficacy for PCOS (AUC = 0.799), and the combined diagnosis of miR-222, miR-146a, and miR-30c can improve diagnostic efficacy (AUC = 0.852) [22]. This study found that serum miR-338-3p has significant diagnostic value for PCOS (AUC = 0.861), providing a new candidate biomarker for non-invasive diagnosis of PCOS. In addition, combined detection of biomarkers may improve diagnostic efficiency, which is worthy of further exploration.

Granulosa cells are essential for follicle development, providing nutrition and support to oocytes and participating in steroid hormone synthesis [23]. Abnormal proliferation and apoptosis of these cells are closely linked to PCOS. The results of this study showed that downregulation of miR-338-3p inhibited cell proliferation and promoted cell apoptosis, while upregulation of miR-338-3p had the opposite effect. This suggests that the regulation of granulosa cells by miR-338-3p may be associated with cell cycle proteins and apoptosis-related proteins. Specifically, reduced miR-338-3p can suppress the cell proliferation via interaction with CyclinD1 [24]. Besides, it can also promote apoptosis by increasing pro-apoptotic proteins Bax and decreasing anti-apoptotic proteins Bcl-2 [25]. These studies highlight the significant regulatory role of aberrantly expressed miR-338-3p in the proliferation as well as apoptosis of granulosa cells. Recent studies have revealed that, in addition to apoptosis, novel forms of programmed cell death such as ferroptosis, cuproptosis, and disulfidptosis also play important roles in PCOS. A study has reported that miR-338-3p affects lipid peroxidation in hepatocellular carcinoma cells by regulating ACSL4 (a key enzyme of ferroptosis) [26], indicating its potential interaction with the ferroptosis pathway. The cuproptosis-related gene SLC31A1 (copper transporter) is upregulated in granulosa cells of PCOS patients. Additionally, glutathione depletion and oxidative stress dependent on disulfidptosis play important roles in insulin resistance of PCOS [27, 28]. Whether these cell death mechanisms may influence the fate of granulosa cells in PCOS, or whether miR-338-3p may interact with these pathways, deserves in-depth exploration.

Furthermore, miR-338-3p may indirectly regulate ovarian function by the insulin signaling pathway. The reduced miR-338-3p in PCOS may be mechanistically linked to key PCOS-associated pathways. Insulin resistance and hyperinsulinemia, central features of PCOS, could suppress miR-338-3p via the PI3K/Akt pathway. PCOS patients often have IR, where hyperinsulinemia inhibits miR-338-3p transcription via PI3K/Akt/mTOR pathway activation. For example, IGF-1 reduces miR-338-3p expression by decreasing transcription factor SP1 binding to its promoter [29]. In hepatocytes, the insulin signaling pathway downregulates miR-338-3p expression by inhibiting the transcription factor SP1 [16], and a similar mechanism may exist in ovarian granulosa cells. Importantly, our study also demonstrated that PTEN is a target gene of miR-338-3p. PTEN is a key regulator that inhibits cell proliferation, differentiation, and apoptosis via suppressing the PI3K/Akt pathway [30]. In our study, PTEN was found to be upregulated in PCOS, and its knockdown significantly improved cell proliferation and inhibited apoptosis. Similar findings have been reported previously. For instance, The PTEN expression is higher in PCOS rat models than in controls [31]. PTEN knockout is also reported to be conducive to reducing PCOS [32]. Additionally, as a negative regulator of the PI3K/Akt pathway, PTEN plays a central role in insulin resistance [33]. In granulosa cells, PTEN upregulation inhibits Akt phosphorylation, impairing insulin-mediated glucose uptake and FSH-induced estrogen synthesis [34]. Based on prior research, we postulate PCOS may upregulate PTEN via miR-338-3p downregulation, suppressing PI3K/Akt signaling and promoting insulin resistance in ovarian cells. In summary, miR-338-3p negatively regulates PTEN, and the upregulation of PTEN is closely related to the occurrence of PCOS. However, the specific mechanism of action remains to be further explored.

The interactions between genes and long non-coding RNAs (lncRNAs) can be investigated within the framework of regulatory networks, a research approach that is increasingly being utilized to understand complex biological systems [35, 36]. Notably, lncRNA MEG3 acts as a molecular sponge for miR-338-3p, promoting granulosa cell apoptosis by relieving the inhibition of PTEN [34]. Additionally, a recent study has revealed that circRNA hsa_circ_0046060 inhibits miR-338-3p expression, leading to upregulation of G6PC2 and exacerbation of insulin resistance [29]. These findings indicate that miR-338-3p may be embedded in a complex lncRNA/circRNA regulatory network, synergistically influencing metabolic abnormalities and follicular development in PCOS.

This study has certain limitations, including the absence of an independent validation cohort, potential impacts of cell line model homogeneity vs. in vivo microenvironmental differences on PTEN expression regulation (future studies will establish primary granulocyte culture systems using clinical samples for validation), unclear regulatory roles of insulin/IGF-1 in miR-338-3p expression, and lack of in vivo animal models to validate its direct regulatory effects on follicular development and hormonal homeostasis.

## Conclusions

Collectively, we found the reduced miR-338-3p showed promising predictive value in distinguishing individuals with PCOS from normal population. Reduced miR-338-3p restrained the proliferation of granulosa cells and enhanced their apoptotic rate by PTEN.

## Data Availability

The datasets used and/or analysed during the current study are available from the corresponding author on reasonable request.
